# Chemical Reactions Directed Peptide Self-Assembly

**DOI:** 10.3390/ijms160510797

**Published:** 2015-05-13

**Authors:** Dnyaneshwar B. Rasale, Apurba K. Das

**Affiliations:** Department of Chemistry, Indian Institute of Technology Indore, Khandwa Road, Indore 452017, India; E-Mail: db.rasale@iiti.ac.in

**Keywords:** chemical reaction, peptide, self-assembly, enzyme, native chemical ligation

## Abstract

Fabrication of self-assembled nanostructures is one of the important aspects in nanoscience and nanotechnology. The study of self-assembled soft materials remains an area of interest due to their potential applications in biomedicine. The versatile properties of soft materials can be tuned using a bottom up approach of small molecules. Peptide based self-assembly has significant impact in biology because of its unique features such as biocompatibility, straight peptide chain and the presence of different side chain functionality. These unique features explore peptides in various self-assembly process. In this review, we briefly introduce chemical reaction-mediated peptide self-assembly. Herein, we have emphasised enzymes, native chemical ligation and photochemical reactions in the exploration of peptide self-assembly.

## 1. Introduction

The spontaneous formation of ordered structures at the nanoscale is usually referred to as self-assembly [1]. When the constitutive components are molecules, the process is generally termed as molecular self-assembly. The molecular self-assembly process is again divided into intramolecular and intermolecular self-assembly. The term molecular self-assembly refers to intermolecular self-assembly and the intramolecular analogue is more commonly called folding. Several studies on the origin of life noted that there must have been processes by which prebiotic organic compounds were sufficiently concentrated [2] to undergo physical and chemical interactions. The physical properties of certain kind of molecules lead to the formation of complex structures with emergent properties. Such emergent phenomena are referred to as self-assembly processes or self-organization. Cellular life began when self-assembled membrane-bound polymers had ability to not only polymerize, but also replicate their linear sequence of monomers [3]. Thus, self-assembly is a basic process by which contemporary cellular life produces membranes, duplex DNA and folding proteins. It has been demonstrated that the first cell must have been formed by the same intermolecular interactions and self-assembled structures. Self-assembly is a prevalent process in nature which plays an important role in maintaining integrity of cells [4,5] to perform various functions of cells [6]. The cellular components such as actin filaments, microtubules, DNA, vesicles and micelles are the classic representation of molecular self-assembly in biological pools [7].

## 2. Development of Molecular Self-Assembly

Molecular self-assembly [8] is the spontaneous association of molecules under equilibrium conditions into stable, structurally well-defined aggregates joined by non-covalent interactions. Molecular self-assembly is a prevalent process in biological systems and underlies the formation of a wide variety of complex biological structures [9,10]. The understanding that the self-assembly process utilizes the association of non-covalent interactions [11,12] of molecular backbones in biological aggregates is a central concern in chemical biology. Besides the biomacromolecular nanostructures, certain small organic molecules are capable to self-assemble in a particular solvent, resulting in self-supporting gel [13,14,15,16,17]. If the self-assembly occurs in an aqueous medium, the resulting gel is referred to a supramolecular hydrogel [18,19]. The design of biomolecules that can self-assemble into higher order structures, have received increasing attention over the past few years, because of their applications in supramolecular electronics [20], drug delivery [21,22,23], wound healing, biosensing [24,25] and tissue engineering [26,27,28]. There are many weak interactions such as hydrogen bonding, hydrophobic interactions and π–π stacking interactions that govern the assembly of everything from DNA in its double helix to the triple helical structure in collagen fibers. Self-assembly is also the only practical approach to build a wide variety of nanostructures [29,30].

**Figure 1:**
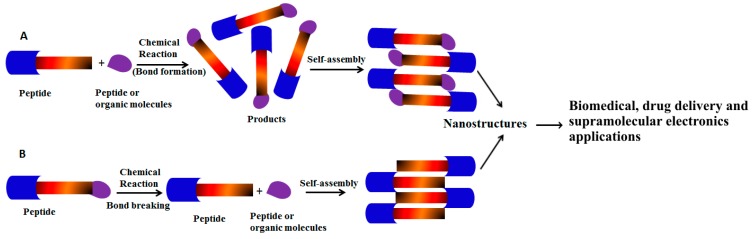
(**A**) Schematic representation shows simple peptide building blocks undergo chemical reactions with peptide or organic molecules to form self-assembled nanostructures via non-covalent interactions; (**B**) Peptides undergo bond breaking via chemical reactions to form peptide self-assembly.

The development of nanoscale structures and devices can be accomplished through “bottom-up approach” or “top-down” methods. In the bottom-up approach, small building blocks assemble into larger structures [31,32] (Figure 1). Examples of this approach include chemical synthesis [33], molecular self-assembly [34], and colloidal aggregation [35,36,37]. Most of the self-assemblies are directed by small molecular weight organic molecules and bioactive molecules. With the increasing applications of supramolecular hydrogels in biomedicine, there is wide interest in the development of supramolecular soft materials [38]. Several, physical stimuli such as pH, temperature, light, enzymes, and sonication are used to control peptide self-assembly.

In this review, we aim to describe various mild chemical reactions in the development of peptide self-assembly (Table 1). A chemical reaction is a process that leads to the transformation of one set of chemical substances to another, which are usually characterized by chemical changes. Chemical reactions are used for the synthesis of new compounds. It has a crucial role not only in everyday life but also in biology where it is referred as metabolism. In living organisms, biochemical reactions are mainly controlled by enzymes. One of the most important biochemical reactions is anabolism, in which different DNA and enzyme-controlled processes result in the production of large molecules such as proteins and carbohydrates from smaller units. There has been wide interest to achieve such processes in the laboratory to develop some complex architecture exhibiting structural complexity ranging from nano- to mesoscale which is of fundamental importance for various protein-related diseases but also holds great promise for various nano- and biotechnological applications [39]. Several physical perturbations are known to develop such complex self-assembled architectures. However, chemical reactions find wide scope in the development of self-assembled biomaterials due to its one pot propensity. Generally, the efforts are made to develop bioorthogonal chemical reactions to further explore in the biomedical applications [40,41]. The self-assembly is mainly governed by non-covalent interactions which could be achieved by adding or removing constraining moieties from the molecules [42]. Here we will emphasize some important chemical reactions in peptide self-assembly.

**Figure 2:**
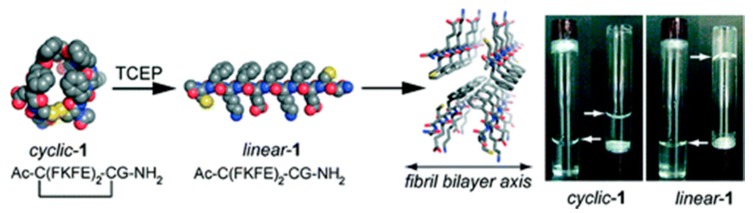
Cyclic to linear peptide conformational switch using a chemical reductive trigger, (adapted from reference [43] with permission from American Chemical Society).

The self-assembly of short peptides can be controlled by imposing a conformational constraint that leads to the prevention of the β-sheet structure (Figure 2) [43]. Nilsson *et al.* flanked a short self-assembling peptide sequence with cysteine (Cys) residues that enabled the macrocyclization of these peptides [43]. Macrocyclization prevents β-sheet formation and self-assembly in the cyclic form. Thus, using TCEP, constraint was removed by simple reduction of the disulfide bond that resulted in relaxation to the stable β-strand and subsequent formation of self-assembly.

**Table 1:** Self-assembly driven by chemical reactions.

Entry	Chemical Reactions	Catalyst/Reaction Condition	Reaction Medium	References
1	Disulfide formation	Air	Aqueous	[43]
2	Photochemical reaction	Light	Aqueous	[44,45,46]
3	Enzymatic reactions	Enzyme	Aqueous	[47,48,49,50]
4	Enzymatic reactions	Enzyme	Organic	[51,52]
5	Thioester mediated native chemical ligation	4-Mercaptophenyl acetic acid	Aqueous	[53,54,55]
6	Oxo-ester mediated native chemical ligation	Heating 80 °C	Aqueous/MeOH	[56,57,58]
7	Seleno ester mediated native chemical ligation	No Catalyst	Aqueous/EtOH	[59]

**Figure 3:**
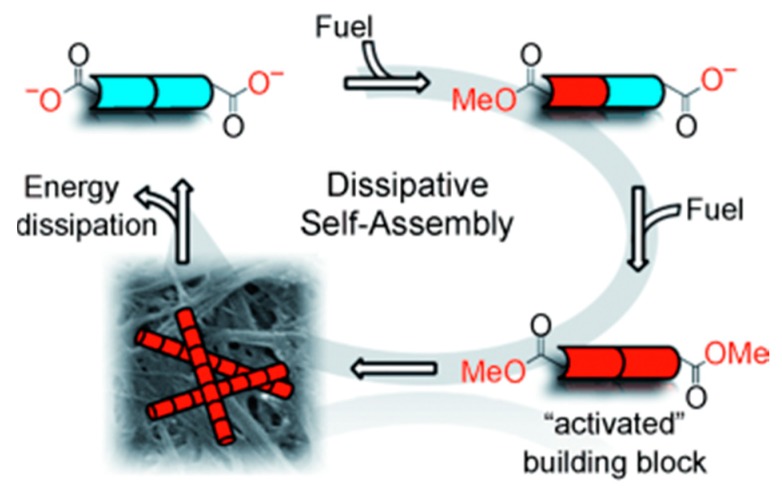
A monomeric building block (blue) is activated by fuel consumption and is able to assemble (forming red fibers). In the assembled state, it can dissipate its energy and revert to its monomeric state (blue), (adapted from reference [60] with permission from John Wiley and Sons).

Van Esch *et al.* reported a dissipative self-assembly system in which a synthetic DSA fibrous network uses chemical fuel as an energy source [60] (Figure 3). A gelator precursor dibenzoyl-(l)-cystine (DBC) is converted into self-supporting gel by reaction with a chemical fuel methyl iodide at pH = 7 leading to the formation of diester. Hydrolysis of the methyl esters of the gelator, which is labile under ambient conditions, leads to energy dissipation and dis-assembly of the formed structures. Yang *et al.* reported redox controllable self-assembly properties of selenium containing peptides [61].

An N-capped 4-phenyl-selenyl butanoic acid peptide was converted to selenoxide upon oxidation with H_2_O_2_, which is easily soluble in phosphate buffer saline [61]. However, chemical treatment with vitamin C converts selenoxide into less soluble selenide in aqueous medium leading to the formation of self-assembling nanostructures. Lehn and coworkers described that guanosine hydrazide yields a stable supramolecular hydrogel based on the formation of a guanine quartet (G-quartet) in the presence of metal cations. Guanosine hydrazide and its assemblies can be reversibly decorated by acylhydrazone formation upon reaction with various aldehydes, resulting in the formation of highly viscous dynamic hydrogels [62]. The dynamic system selects an aldehyde from the mixture of aldehydes, which leads to the formation of the most stable gel. Rao *et al.* demonstrated a biocompatible condensation reaction for controlled assembly of nanostructures in living cells using 1, 2 aminothiol and 2-cyanobenzothiazole [63]. Ajayan reported uniform and crystalline nanofibers of perylene-3,4,9,10-tetracarboxylic dianhydride (PTCDA), an insoluble organic semiconducting molecule which have been achieved by self-assembling molecules using chemical reaction mediated conversion of an appropriately designed soluble precursor perylene tetracarboxylic acid (PTCA) using carbodiimide chemistry [64]. Das *et al.* exploited a reversible esterification reaction that leads to the formation of a single predominant product among the library members using dimethyl sulfate (DMS) as chemical fuel [65]. The library members formed a self-supporting hydrogel and showed the formation of a single predominant product. Otto and coworkers developed two self-replicating peptide-derived macrocycles that emerge from a small dynamic combinatorial library through oxidative disulfide formation from their pendant thiol groups in presence of oxygen and compete for a common feedstock. Replication is driven by nanostructure formation resulting from self-assembly of peptide [66].

## 3. Photo-Switched Molecular Self-Assembly

**Figure 4:**
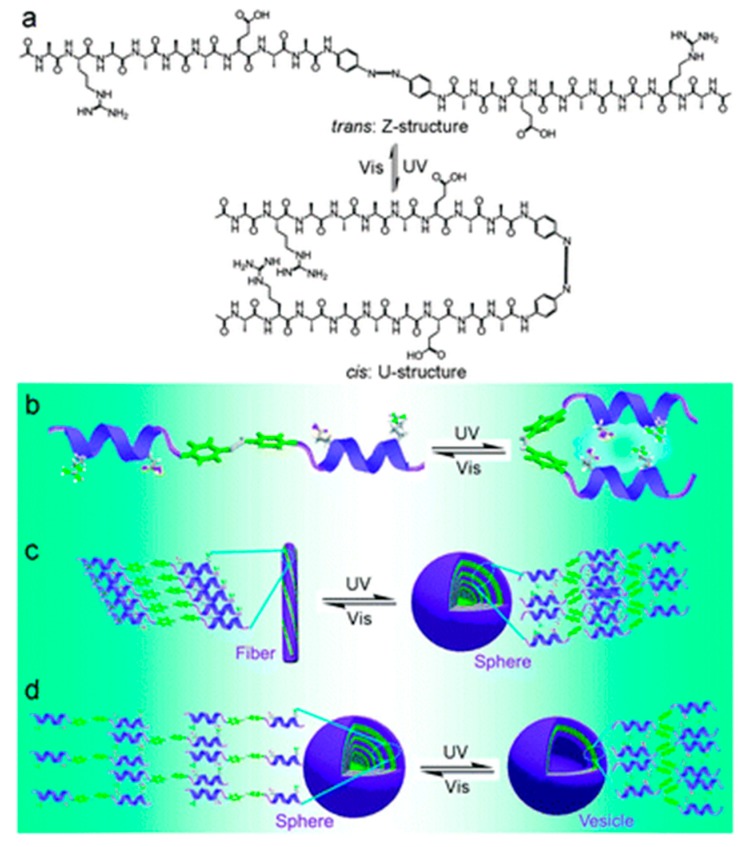
Schematic illustration of the light-switched self-assembly of the gemini α-helical peptide. (**a**) Molecular structure of the gemini α-helical peptide and light-triggered reversible change between Z- and U-structures; (**b**) Model of light-triggered reversible structural change in the gemini α-helical peptide; (**c**) Light-switched self-assembly behaviors of the gemini α-helical peptide in acidic (pH 3.0); and (**d**) in basic (8.0) medium, (adapted from reference [44] with permission from Royal Society of Chemistry).

The self-assembly of bio-organic molecules into nanostructures is an attractive route to fabricate functional materials. For example, diphenylalanine (Phe-Phe, FF), an aromatic dipeptide consisting of two covalently linked phenylalanine units, can form various nanostructures such as nanotubes [67,68], nanowires and nanosphere [69] under different processing conditions [70]. FF can readily self-assemble into different nanostructures in a simple way and possess the functional flexibility and molecular recognition capability suitable for a wide range of applications, such as biosensors [71], imaging, guest encapsulation and nanofabrication [72,73,74]. Zhang *et al.* reported an azobenzene-linked symmetrical gemini α-helical peptide which reversibly transforms between the *trans*- (Z-) and *cis*-structure (U-structure) under UV (λ = 365 nm) and subsequently visible light irradiation [44] (Figure 4). This also affects self-assembly behavior of the gemini α-helical peptide. Park *et al.* developed light-harvesting peptide nanotubes that integrate photosynthetic units for mimicking natural photosynthesis [45]. Zinic *et al.* demonstrated that a bis(phenylalanine) maleic acid shows irreversible photoinduced gelation in water that works on photochemical isomerization of nongelling maleic acid amide to gelling fumaric acid amide [46].

## 4. Enzyme Catalyzed Peptide Self-Assembly

**Figure 5:**
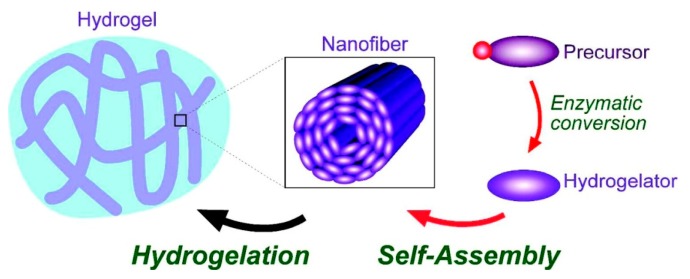
The essential steps in the enzymatic hydrogelation of small molecules, (adapted from reference [78] with permission from American Chemical Society).

Enzymes are a class of highly efficient and specific catalysts in nature. Enzyme-regulated molecular self-assembly plays a critical role in many cell processes [47]. The formation of microtubules, which governs mitosis, is one example [75]. The polymerization of actins which governs the focal adhesion of cells, is essentially an enzyme regulated self-assembly process of seemingly miraculous sophistication. These natural self-assemblies inspire the development of enzymatic hydrogelation of small molecules. Compared with physical or conventional chemical perturbations, enzymatic regulation promises a unique opportunity to integrate molecular self-assembly in water with natural biological processes. Moreover, as a new method to make biomaterials, the enzyme-catalyzed formation of hydrogels of small molecules has already shown promise in biomedical applications [48,49]. Xu and coworkers reported a method to image enzyme-triggered self-assembly of small molecules inside live cells [76]. George *et al.* have focused on the enzyme catalyzed self-assembly strategy to develop molecularly defined and functional materials [77]. Ulijn *et al.* exploited several enzymes including phosphatases, esterases and proteases, to trigger the self-assembly of aromatic peptide amphiphiles by converting non-assembling precursors into self-assembling components [78,79,80,81] (Figure 5). Saiani *et al.* reported the effect of enzyme concentration on the morphology and properties of enzymatically triggered peptide hydrogels [82].

## 5. Self-Assembly Driven by Peptide Hydrolysis

Generally, hydrolysis is a chemical process in which a molecule of water is added to a substance. This addition causes both substance and water molecule to split into two parts. Acid-base-catalysed hydrolyses are very common. One such example is the hydrolysis of amides or esters [83]. The amide bond in a peptide is more rigid due to significant delocalisation of the lone pair of electrons on the nitrogen atom giving the bond a partial double bond character. Therefore, the hydrolysis reactions are very slow in water. Usually in the laboratory, hydrolysis of proteins or peptides is being carried out by using 6 N HCl at 110 °C [84]. However, use of this method leads to partial destruction of many amino acids including serine, threonine and cysteine. Tryptophan is totally destroyed by this procedure. In a living system, most biochemical reactions including ATP hydrolysis take place by the catalysis of enzymes. The catalytic action of enzymes allows the hydrolysis of proteins, fats, oils, and carbohydrates. Protease enzymes aid digestion by causing hydrolysis of peptide bond in proteins [85]. In recent years, there has been a growing interest in the development of peptide nanostructures using biocatalytic methods via peptide bond hydrolysis. Researchers are interested to explore enzymatic development of peptide self-assembly for further biomedical applications without destruction of any amino acids. Shao *et al.* reported self-assembly of a peptide amphiphile based on hydrolysed Bombyx mori silk fibroin usin α-chymotrypsin [50]. Das *et al.* described a general strategy to control the state of molecular self-assembly under thermodynamic control using the protease thermolysin. Self-assembly drives the formation of a single π-stacked predominating product in a dynamic library [86].

**Figure 6:**
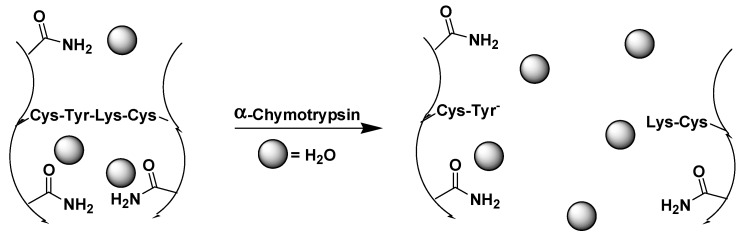
A CYKC-cross-linked hydrogel degraded with α-chymotrypsin, (adapted from reference [87] with permission from American Chemical Society).

Moore and coworkers described a chymotrypsin responsive hydrogel in which the tetrapeptide CYKC was used as a cross-linker to create a poly(acrylamide) hydrogel [87] (Figure 6); the sequence of CYKC hydrolysis by chymotrypsin leads to degradation of the hydrogel.

## 6. Peptide Self-Assembly Driven by Amide Bond Formation

Amide bond formation of peptides is exactly opposite to the hydrolysis of amide bonds in peptides. Usually some proteases show the ability to reversibly synthesize the amide bond as well as hydrolyze the amide bond in dilute aqueous conditions. It has been reported that self-assembly drives the reverse hydrolysis of peptides and prefers the most stable product formation in gel phase medium*.* Thus, the enzyme thermolysin is preferably used to exploit peptide self-assembly. Self-assembly of macroscopic materials from small molecular building blocks provides a route to design molecular biomaterials. The controlled development of biomaterials is in high demand in the context of biomedical applications. The stimuli that trigger self-assembly include various physical chemical perturbations. Enzyme triggered self-assembly is particularly interesting due to mild and physiological reaction conditions. Enzymes allow biocatalytic reactions in a biological environment which could lead to hydrogelation of peptides. Herein, some examples demonstrate the hydrogelation of peptides via reverse hydrolysis of amide bonds in peptides.

**Figure 7:**
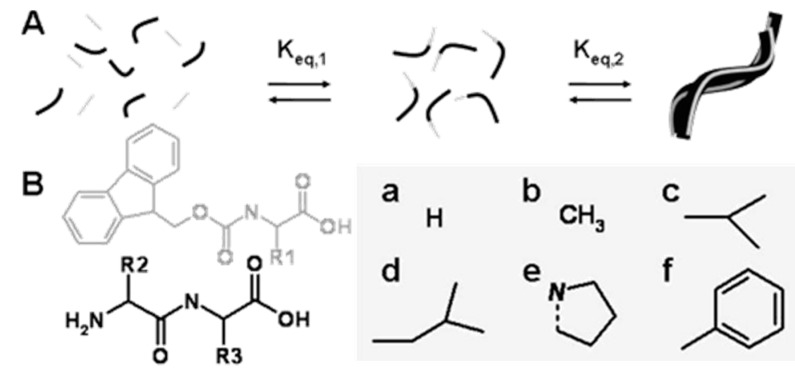
(**A**) Proposed mechanism: Fmoc amino acids (gray) are enzymatically coupled to dipeptides (black) by a protease to form Fmoc-tripeptides that self-assemble to higher-order aggregates driven by π-π interactions between fluorenyl groups. K_eq,1_ represents the equilibrium constant for peptide synthesis/hydrolysis, K_eq,2_ for self-assembly; (**B**) Chemical structures of Fmoc-amino acids, dipeptide precursors and amino acid side chains: a Gly, b Ala, c Val, d Leu, e Pro, f Phe, (adapted from reference [88] with permission from American Chemical Society).

Ulijn *et al.* have used a protease enzyme that normally hydrolyzes peptide bonds in aqueous medium. They described a conceptually novel approach by using thermolysin to perform the reverse reaction (*i.e.*, peptide synthesis or amide formation), which can produce amphiphilic peptide hydrogelators and self-assembles to form nanofibrous structures [88] (Figure 7). The Ulijn group also reported the use of reversible enzyme-catalysed reactions to drive self-assembly. They demonstrated that this system combines three features such as (i) self-correction-fully reversible self-assembly under thermodynamic control; (ii) component-selection ability to amplify the most stable molecular self-assembly structures in dynamic combinatorial libraries and (iii) spatiotemporal confinement of nucleation and structure growth [89]. Enzyme-assisted self-assembly therefore provides control in bottom-up fabrication of nanomaterials that could ultimately lead to functional nanostructures with enhanced complexities and fewer defects. Das *et al.* reported bio-catalytic evolution of dynamic combinatorial libraries via amide bond formation. Self-assembly evolves thermodynamically downhill library members via enzyme-catalysed amide bond formation [90].

## 7. Lipase Catalyzed Peptide Self-Assembly

Lipase is an important class of enzyme that catalyses the hydrolysis of fats (lipid). Lipases are the most broadly deployed biocatalysts because of their ability to produce chiral products [91] with high enantiomeric purity. Lipases are used as catalysts for hydrolysis, alcoholysis, esterification and transestrification of carboxylic acids or esters reactions [92,93]. They also work in organic as well as in aqueous medium, which makes it a versatile candidate in the broad research area. One of the important aspects in chemical synthesis of drugs is to retain a single enantiomer, which is often a difficult task. Thus, one of the commercially attractive and environmentally compatible ways of making enantiomerically pure drugs is biotransformation. In addition, peptides acylated with fatty acids become capable of being anchored to liposomes, translocating across lipid membranes, penetrating intact cells, and penetrating through the blood-brain barrier. However, selective acylation is a formidable task to a chemist due to the presence of numerous reactive groups in peptides. Klibanov *et al.* reported a lipase catalyze selective acylation of a dipeptide l-Phe-α-l-Lys-O^t^Bu [94]. It has two primary amino groups. The α-NH_2_ group of Phe and the ω-NH_2_ group of Lys offer a challenge for selective acylation. Thus, lipase selectively acylates the ω-NH_2_ group of Lys in dipeptides in the presence of excess trifluoroethyl acetate. Besides its useful applications in organic synthesis, lipases are being used in the development of molecular self-assembly. Recently, the use of enzymes for the fabrication of biomaterials, starting from small molecular building blocks, to promote the synthesis of self-assembling materials has become an emerging area of research activity. Additionally, the structure of these (bio) materials can be easily controlled and tuned by using biological catalysts taking advantage of chemo-, regio- and enantioselective synthesis and of mild reaction conditions. In general, there are two routes to drive enzymatic molecular self-assembly through either breaking or making of covalent bonds. Interestingly, both routes lead to self-assembly by maintaining the hydrophobic and hydrophilic balance between the self-assembling molecules. Important efforts have been made to gain more insight into molecular self-assembly.

**Figure 8:**
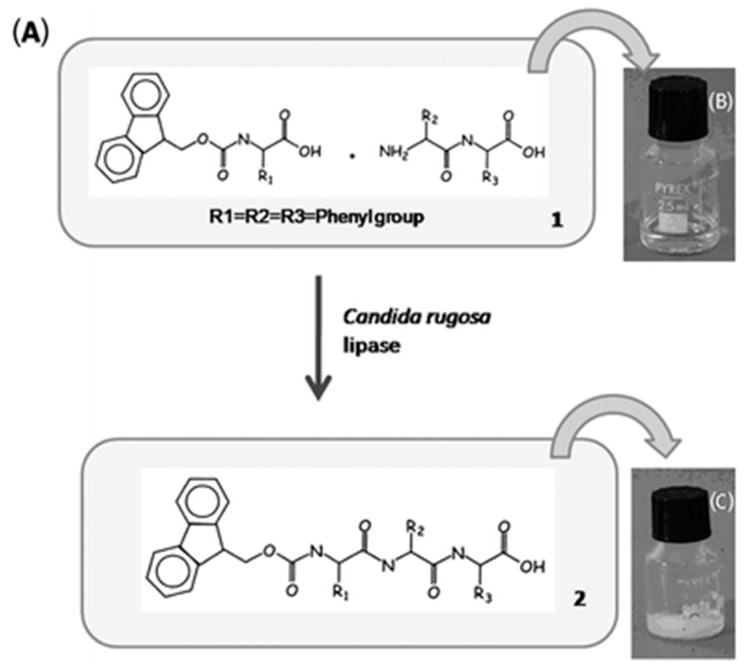
(**A**) Chemical structures of the precursor (1) and its corresponding hydrogelator (2) and the schematic gelation process; (**B**) optical images of a solution of 1 in phosphate buffer (pH 7.4) and (**C**) the hydrogel of 2 formed by adding lipase to a solution of 1, (adapted from reference [95] with permission from Royal Society of Chemistry).

These studies have proved that non-covalent interactions such as π-π stacking, hydrogen bonding and hydrophobic interactions play a key role in the development of such systems. Recently, thermolysin was used to generate dynamic combinatorial libraries for the discovery of stable self-assembling nanostructures. Although lipases are primarily used in esterification and transesterification of carboxylic acids, there are few examples where lipases have been used to develop peptide self-assembly through amide bond synthesis. Palocci and colleagues reported peptide self-assembly via coupling of Fmoc-phenylalanine and diphenylalanine using lipase as the catalyst at physiological conditions [95] (Figure 8). This is an excellent example of synthesis of a peptide bond instead of using expensive protease. Lipase is known as an industrial biocatalyst and can be exploited to study such self-assembling systems. In another work, the Palocci group described the self-assembly of homochiral and heterochiral lipase catalyzed Fmoc-based peptides with control drug release from hydrogel metrix [51].

**Figure 9:**
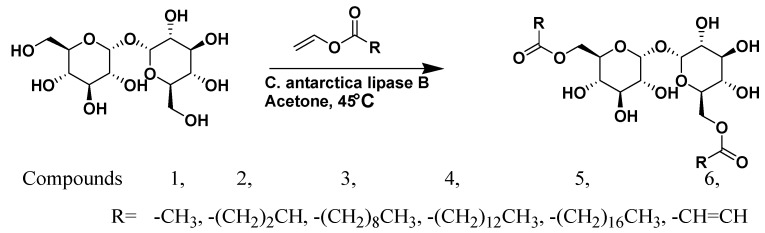
Lipase catalyzed acylation of the disaccharide trehalose generated a family of low-molecular-weight gelators via transesterification, (adapted from reference [52] with permission from John Wiley and Sons).

Dordick *et al.* demonstrated lipase catalyzed sugar-containing self-assembled organogels with nanostructured morphologies [52] (Figure 9). The lipase activity and thermostability can be increased upon immobilization with many supports. The self-assembled peptide architectures has also been used for the improved activity of lipase. Matsui *et al.* reported lipase incorporated peptide nanotubes [96]. However, there are some nonspecific proteases that have been used for ester hydrolysis followed by self-assembly. Although, lipases are widely used in ester hydrolysis and transesterification, it is less explored in molecular self-assembly. Das *et al.* described lipase-catalysed incorporation of gastrodigenin (*p*-hydroxybenzyl alcohol) to Nmoc-protected peptides [97]. The lipase catalysed esterification reaction results in the formation of blue light emitting peptide nanofibers in aqueous medium. Self-assembly of peptides evolves blue light emission upon illumination under UV light. They also reported that *p*-hydroxybenzyl alcohol can efficiently be incorporated into peptide bolaamphiphiles [98]. The activated esterified products self-assemble to form thixotropic hydrogel. A self-assembled hydrogel matrix was used for 3D cell culture. Significant cell support and proliferation of human umbilical cord mesenchymal stem cells were observed.

## 8. Native Chemical Ligation

**Figure 10:**
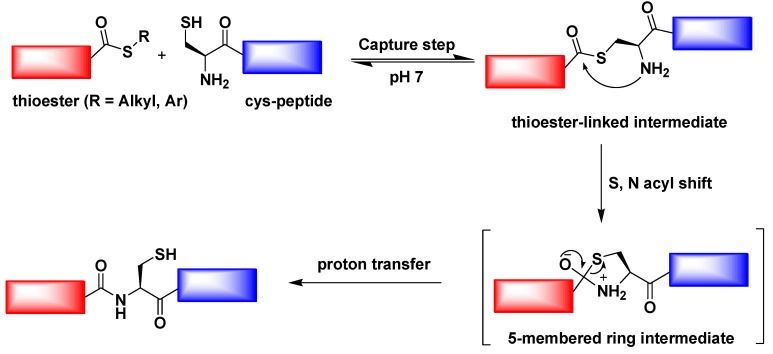
Native chemical ligation (NCL) mechanism.

Native chemical ligation (NCL) is an important extension of the chemical ligation discovered by Kent and co-workers in 1994 [99]. It is a widely used method for the total or semi-synthesis of proteins. Native chemical ligation involves the chemoselective reaction of two unprotected peptides in aqueous solution to give a single covalently linked ligated product (Figure 10). In fact, in 1953, Wieland and co-workers discovered the chemical foundation of this reaction [100]. The reaction of valine-thioester and cysteine amino acid in aqueous buffer was shown to yield the dipeptide valine-cysteine. The reaction proceeds through the intermediacy of a thioester containing the sulfur of the cysteine residue. Wieland’s work led to the “active ester” method for making protected peptide segments in conventional solution synthesis in organic solvents.

## 9. Peptide Self-Assembly Driven by Thioester Mediated Native Chemical Ligation

As posited in a “Thioester World”, thioesters are possible precursors to life [101]. It is revealed that thioesters are obligatory intermediates in several key processes in which ATP is either used or regenerated. Thioesters are involved in the synthesis of all esters, including those found in complex lipids. The biosynthesis of lignin, which comprises a large fraction of biomass, proceeds via thioester derivative of caffeic acid. Acetyl CoA is an important molecule in metabolism. The chemical structure of Acetyl CoA includes a thioester between coenzyme A (a thiol) and acetic acid that is produced during the second step of aerobic cellular respiration in the Krebs cycle. The *C*-terminal peptides or protein thioesters are essential in NCL. The synthetic thioester preparation is generally carried out via conventional carboxyl activation chemistry rather than the N→S route so elegantly directed by intein. Thioesters can also be prepared via *tert*-butyloxycarbonyl (Boc)-based solid-phase peptide synthesis (SPPS) on a thioester resin or 9-fluorenylmethoxycarbonyl (Fmoc)-based SPPS, which involves mild bases during synthesis [102]. Melnyk and co-workers reported a solid-phase N→S acyl transfer for thioester synthesis after peptide chain assembly using Fmoc/t-Bu chemistry in combination with the sulfonamide safety-catch linker [103]. Once, the thioester is synthesized, it can easily be subjected to NCL reaction at physiological conditions. Since the beginning of thioester-mediated NCL reactions, it has been exploited for total synthesis of proteins or peptides and dendrimers [53] and is combined with peptide self-assembly. Recently, NCL reactions have attracted broad attention from research groups for the development of functional biomaterials.

**Figure 11:**
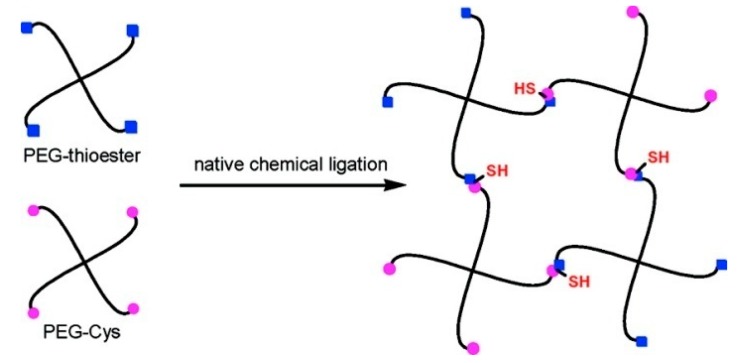
Native chemical ligation is used in cross-linking of hydrogel, (adapted from reference [104] with permission from American Chemical Society).

Woolfson *et al.* described the combination of chemical ligation with peptide self-assembly to deliver extremely long polypeptide chains with stipulated, repeated sequences. The self-assembling fibers were used to align peptide from their *N-* to *C*-terminals, which facilitates the ligation reactions without the usual requirement of an *N*-terminal catalytic cysteine residue [54]. Collier and co-workers investigated a novel method for rapidly increasing the stiffness of self-assembled β-sheet fibrillar peptide hydrogels using native chemical ligation (NCL) [55]. Messersmith *et al.* illustrated the use of NCL as a strategy to form covalently cross-linked polymer hydrogels under mild conditions and in the absence of catalysts [104] (Figure 11). The thioester based polymer and *N*-terminal cysteine polymer bioconjugate were synthesized to bring up hydrolgelation by native chemical ligation reaction and the viscoelastic nature of hydrogel was studied by oscillatory rheology.

## 10. Peptide Self-Assembly Driven by Oxo-Ester Mediated Native Chemical Ligation

**Figure 12:**
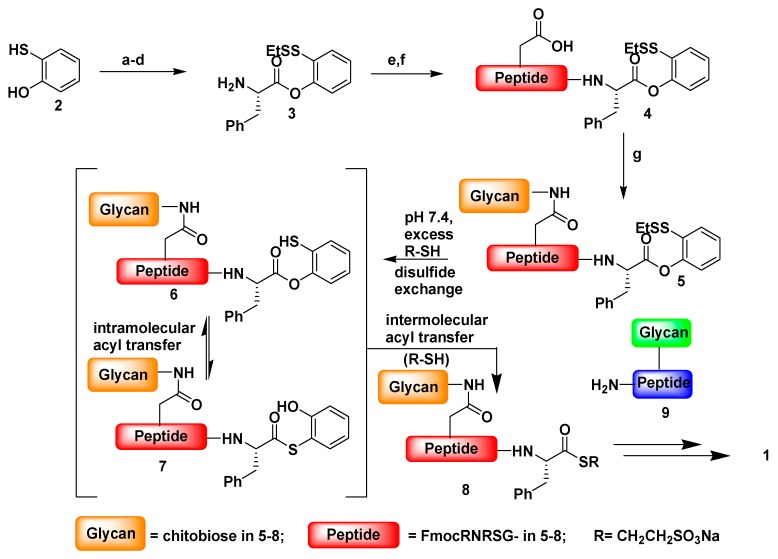
Oxo-ester mediated native chemical ligation, reagents and conditions: (a) I2, MeOH, H2O; (b) BF3-OEt2, EtSSEt, CH2Cl2, 99%, 2 steps; (c) Boc-Phe-OH, EDCI, DMAP, CH2Cl2/THF, 93%; (d) 4 N HCl/dioxane, 94%; (e) Fmoc-Arg(Pbf)-Asp(tBu)-Arg(Pbf)-Ser(tBu)-Gly-OH, HATU, DIEA, DMF, 61%; (f) TFA/phenol/Et3SiH/H2O, 35:2:1:1, 60%; (g) GlcNAcβ1→4GlcNAcβ1-NH2, HATU, DIEA, DMSO, 52%, (adapted from reference [105] with permission from American Chemical Society).

Amino acid and peptide oxo-esters have played an important role in peptide chemistry for many years. Various amino acid esters are commonly used as protecting groups for peptide synthesis in conventional solution phase methodology. Moreover, activated phenyl ester derivatives have been used for chemical ligation of peptides. Several modifications of thioester mediated NCL reactions have been made in an effort to expand the utility of the method. Danishefsky *et al.* first reported the use of oxo-ester in NCL reactions through indirect approach involving o-thiophenolic ester [105], which was followed later by a direct approach utilizing *p*-nitrophenyl (pNP) activated *C*-terminal ester (Figure 12). The oxo-ester mediated native chemical ligation reactions were successfully carried out with sterically hindered *C*-terminal amino acids which is rather problematic with thioester mediated NCL. Hackeng *et al.* observed that β-branched amino acids such as Thr, Val and Ile in this position react extremely slowly under standard NCL reaction conditions (>48 h) [106]. Long NCL reaction times are generally discouraged, due to potential side-reactions (thioester hydrolysis, desulfurization of cysteine and methionine oxidation) [107] under the NCL conditions employed. Moreover, proline in this position was found to react even more sluggishly with, at best, conversions of around 20% after 48 h [108,109]. However, the *p*-nitrophenol oxo-esters are more labile acyl donors than thioester in NCL reactions.

**Figure 13:**
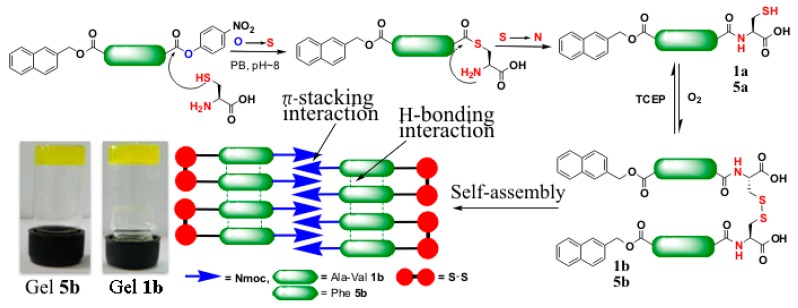
Native chemical ligation at Nmoc-protected-p-NP esters. The cysteine amino acid induces O-S exchange with Nmoc-protected-*p*-NP 1 or 5 (step I) to form a thioester intermediate. Subsequent S-N acyl transfer furnishes the peptide bond 1a or 5a (step II). Air oxidation provides the formation of ligated disulfide 1b or 5b (step III) resulting in supramolecular peptide gels, (adapted from reference [58] with permission from Royal Society of Chemistry).

Synthesis of *p*-nitrophenol ester is easy and less problematic. Weissenborn *et al.* described oxo-ester mediated NCL on oxo-ester activated surfaces and found that 2,3,4,5,6-pentafluorophenyl (PFP) is a more efficient acyl donor than *p*-nitrophenol and *N*-hydroxysuccinimide (NHS) activating agents [110]. Liu *et al.* reported a simple and less activated phenyl oxo-ester of peptide for chemoselective NCL reactions [56]. Borner *et al.* investigated peptide-guided assembly of poly(ethyleneoxide)-peptide conjugate via intramolecular O→N acyl transfer to restore native amide bonds [57]. Our group investigated the role of active *p*-nitrophenyl esters in peptide self-assembly via native chemical ligation [58]. We synthesized Nmoc-protected amino acids/peptides having *C*-terminal *p*-NP esters. The modified ligation precursors undergo self-assembly via NCL with *N*-terminal cysteine residues. Self-assembly via NCL was studied with Nmoc-protected amino acid and peptide *p*-NP esters. Five compounds 1–5 (Figure 13) were synthesized by conventional solution phase methodology.

**Figure 14:**
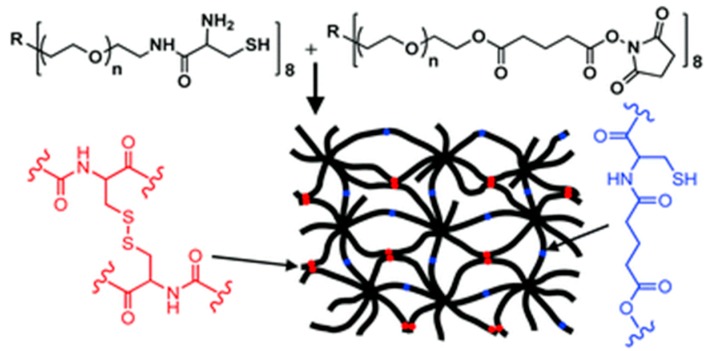
Peptide self-assembly via oxo-ester mediated native chemical ligation, (adapted from reference [111] with permission from Royal Society of Chemistry).

These kinds of soft biomaterials can be used for cell culture, tissue engineering and supramolecular electronics applications. Messersmith *et al.* described polymer hydrogel formation via oxo-ester mediated NCL between branched polymer precursors containing NHS activated ester and *N*-terminal cysteine group and showed cytocompatibility and *in vivo* acute inflammatory response [111] (Figure 14).

## 11. Peptide Self-Assembly Driven by Selenoester Mediated Native Chemical Ligation

**Figure 15:**
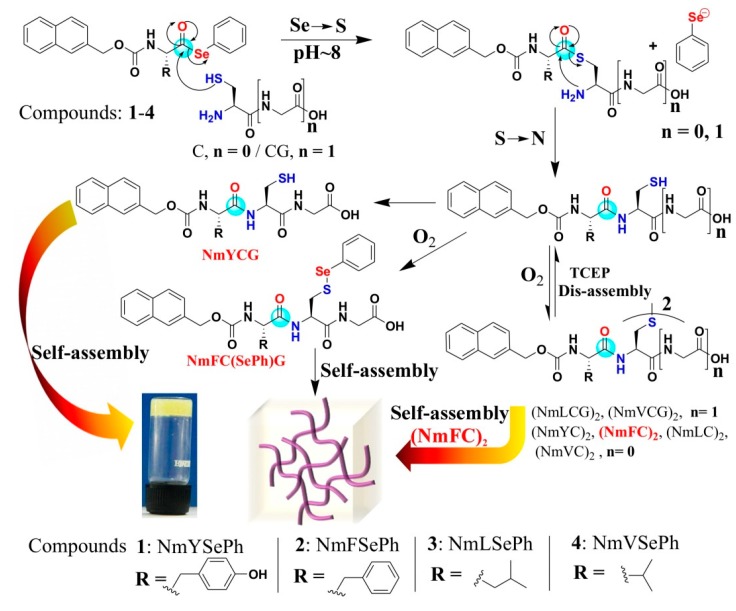
Selenoester mediated native chemical ligation. Ligated products were formed upon the NCL of selenoesters 1-4 with Cys-Gly and cysteine at pH~8. NmYCG self-assembled in its reduced form while oxidized NmFC(SePh)G (sulfur linked with selenophenol) and (NmFC)_2_ self-assembled to form self-supporting soft materials, (adapted from reference [59] with permission from Royal Society of Chemistry).

Over the past decades, selenium and organoselenium compounds are gaining increasing attention due to their properties as antioxidant and antitumor agents, as apoptosis inducers, and in the effective chemoprevention of cancer in a variety of organs [112,113,114,115]. Selenoesters are important intermediates in several organic transformations. The compounds in this class have been used as precursors of acyl radicals [116,117,118] and anions [119] and have attracted attention for the synthesis of new molecular materials, especially superconducting materials, liquid crystals and self-assembled biomaterials. The hydrolysis of selenoesters subsequently generates ionic species of selenium (such as selenols), which can readily participate in redox processes. These charged seleno compounds may possess inherent biological activity, and it would be beneficial if they could enhance the cytotoxic impact on cancer cells. In the case of aryl selenoester, the magnitude of selenoesters hydrolysis as well as its expected biological activity can be tuned by the replacement of different substituent on the aryl ring. The synthesis of selenoesters can be achieved by solution phase methodology as well as Boc solid phase peptide synthesis in peptide chemistry. The reactivity of selenoesters was found higher than the comparable thioesters towards thiol nucleophiles in the first transesterification step of NCL. Therefore, selenoesters have been used for the synthesis of proteins by chemical ligation [120,121], the synthesis of substrates that undergo facile and efficient radical decarbonylation, and the synthesis of natural alkaloid (+)-geissoschizine [122]. Beside the useful application of selenoesters in the above area, there is significant opportunity to explore the selenoesters in the self-assembly process. We have efficiently explored a new method for peptide self-assembly via selenoester-mediated native chemical ligation [59] (Figure 15). In this work, our objective was to develop a simple and efficient method that can direct dynamic peptide self-assembly. To achieve this goal, we synthesized four compounds with an *N*-terminal capped with an aromatic naphthalene-2-methoxycarbonyl (Nmoc) group. The *C*-terminals of 1–4 were protected with phenyl selenoester, which could readily undergo NCL reaction at room temperature with *N*-terminal cysteine and *N*-terminal cysteine based peptide Cys-Gly. Considering the active role of Cys-Gly in increased risk of women breast cancer, we have used Cys-Gly to ligate with Nmoc-protected selenoesters.

The NCL reaction proceeds through the thioester-linked intermediate where acyl transfers from Se→S [123] are followed by intramolecular S→N acyl transfer to give a peptide bond monitored by reverse phase high performance liquid chromatography. Native chemical ligation reaction with selenoesters was very fast and occurred within five minutes. Self-assembly was studied upon native chemical ligation reactions.

## 12. Application of Self-Assembled Materials

There are many applications where self-assembled peptide nanostructures could play an important role as part of biosensing platforms, as efficient drug-delivery systems, cell cultures or as a hydrogels for tissue reparation. Like some of the traditional antibiotics, the short cationic antimicrobial peptides can kill the microbes by interacting and disrupting bacterial cell membranes. Effectiveness of antimicrobial activity depends on the cationic charges and the hydrophobicity of peptides [124]. The self-assembled architectures in associated gel networks and antimicrobial activities of peptide amphiphiles make them potential candidates as cell culture matrices or scaffolds in tissue engineering and regenerative medicine. Extensive study of these self-assembled biomaterials has already proved their biocompatibility [125,126]. Several groups have reported that molecular hydrogels have been widely used as carriers for the delivery of therapeutic agents [127,128]. Self-assembling peptide amphiphiles also have great potential as templates for nanofabrication such as in biomineralization [129,130,131], nucleation, nanowires, and nanocircuits [132]. The self-assembled nanofibers have also been used as templates for the nucleation and growth of CdS nanocrystals [133].

## 13. Conclusions

This review has aimed to provide readers with comprehensive details about peptide self-assembly via mild chemical reactions and various chemical reactions utilized by different research groups for the construction of self-assembled nanostructures in aqueous medium were discussed. Self-assembly is an important process in bottom-up nanotechnology and various covalent and non-covalent interactions govern the self-assembly process. Chemoselective native chemical ligation offers a novel approach for fabrication of self-assembled architectures. *In situ* formation of self-assembled soft materials has significant importance in biology, and peptide based self-assembly is biocompatible and rapid, which allows gelation at physiological conditions to make it an ideal biomaterial.
